# Chinese Herbal Medicines as Potential Agents for Alleviation of Heat Stress in Poultry

**DOI:** 10.1155/2017/8208261

**Published:** 2017-10-31

**Authors:** Parisa Shokryazdan, Mohammad Faseleh Jahromi, Salwani MD Saadand, Mahdi Ebrahimi, Zulkifli Idrus, Hailong Zhou, Xiao Ping Diao, Juan Boo Liang

**Affiliations:** ^1^Institute of Tropical Agriculture, Universiti Putra Malaysia (UPM), 43400 Serdang, Selangor, Malaysia; ^2^Agriculture Biotechnology Research Institute of Iran (ABRII), East and North-East Branch, P.O. Box 91735 844, Mashhad, Iran; ^3^Halal Research Institute, Universiti Putra Malaysia (UPM), 43400 Serdang, Selangor, Malaysia; ^4^Faculty of Veterinary Medicine, Universiti Putra Malaysia (UPM), 43400 Serdang, Selangor, Malaysia; ^5^Department of Animal Science, College of Agriculture, Hainan University, Haikou 570228, China

## Abstract

Heat stress negatively affects the productivity of chickens in commercial poultry farms in humid tropics. In this study, the concentrations and types of the antioxidant compounds of eight Chinese herbal medicines, which have previously demonstrated promising effects on suppressing heat stress as a mixture, were investigated using reversed-phase High Performance Liquid Chromatography, spectrophotometry, Liquid Chromatography Mass Spectrometry, and Gas-Liquid Chromatography. Our results provided the levels of phenolic compounds, total amounts of sugars, and total unsaturated fatty acids in the herbal extracts. Apart from the detection and quantification of the active ingredients of herbs that have the potential to mitigate heat stress in poultry, results of this study also provide useful data for developing an efficient and accurate formulation of the herbs' mixtures in order to induce positive effects against heat stress in* in vivo* studies.

## 1. Introduction

In tropical regions, high environmental temperature in combination with high humidity causes heat stress in chickens, especially in high producing birds that are characterized by high feed intake and metabolic rates, leading to huge economic problems in commercial poultry farms. Heat stress will reduce feed intake, which in turn will adversely affect nutrient uptake and utilization, resulting in decreased productivity rates. Moreover, heat stress induces oxidative stress in the body, and oxidation of substrates produces free radicals and other reactive oxygen species that can cause serious damage to the cells and organs [1]. However, it is well known that antioxidants can be used for elimination or mediation of the adverse effects of heat stress. A biological antioxidant is defined as “any substance that, when present at low concentrations compared to those of an oxidizable substrate, significantly delays or prevents oxidation of that substrate” [2]. Although there are claims that traditional herbal medicines including Chinese herbal medicines (CHM) are effective in reducing adverse effect of heat stress due to their antioxidant activity, scarce data exist describing these effects. Zhang et al. [3] reported that dairy cows fed a diet supplemented with a Chinese herbal formula increased their milk yield by 14%. To support the above positive effect on milk yield, the authors reported that supplementation with the above herbal formula also increased the total antioxidant capacity (T-AOC) of heat stressed cows by 44% compared to the controls (non-heat stressed cows). In a recent study, our research team [4] reported that supplementation with different dosages (0, 0.5, 1.5, and 2%) of a mixture of eight CHM in the diet of laying hens under heat stress conditions (average temperature of 30.1 ± 2.7°C and mean relative humidity of 80.2 ± 7.8%) significantly increased egg production rate, serum antibody titer for New Castle's disease virus, and the superoxide dismutase levels compared to the nonsupplemented birds. At the same time, the malondialdehyde level (as an indicator for oxidative stress) was significantly lower in CHM treated hens compared to the controls. The positive results derived from the above studies suggest that CHM could serve as a potential effective substitute of the conventional antibiotic related drugs commonly used to suppress stress in commercial poultry production. However, the active compounds of CHM that protect animals against heat stress were not determined in the previous studies of Diao et al. [4] and Zhang et al. [3]. The present study, therefore, aimed to provide some insights into the concentrations and types of the active compounds (phenolic compounds, oligosaccharides with potential prebiotic effects, and unsaturated fatty acids) in the eight CHM that possibly play an important role in alleviating the effects of heat stress. In addition, results obtained from this study may also provide new information to develop a more efficient CHM formulation for stress mitigation in farm animals.

## 2. Materials and Methods

### 2.1. Samples

The eight Chinese herbal medicines used in this study were* Chai Hu* (*Bupleurum*),* Bai Zhu* (*Atractylodes*),* Bai Shao* (*Paeonia*),* Gan Cao* (*Glycyrrhiza*),* Bu Gu Zhi* (*Psoralea*),* Bo He* (*Mentha*),* Fu Ling* (*Poria*), and* Shi Gao* (*Gypsum*). CHM were supplied by the College of Agriculture, Hainan University. All the herbs were ground and sieved through 60–80 meshes (particle size 0.32–0.42 mm).

### 2.2. Determination of Phenolic Compounds Using HPLC

The concentrations of phenolic compounds in the herbs were measured by reversed-phase High Performance Liquid Chromatography (HPLC). Phenolic compound standards consisted of gallic acid, vanillic acid, caffeic acid, synergic acid, orientin, sinapic acid, isoorientin, coumarin acid, and* p*-anisic acid. Twenty-microliter methanolic extract from each herbal medicine was loaded on the HPLC instrument (Waters-e2695 series, US) equipped with a UV-Vis photodiode array (PAD) detector, binary pump, vacuum degasser, autosampler, and analytical column (COSMOSIL 5C18-AR-II Packed Column, 4.6 mm I.D. × 250 mm, Japan). Solvents were deionized water and acetonitrile. The pH of water was adjusted to 2.5 with orthophosphoric acid. The phenolic compounds were detected at 280 nm. The column was equilibrated with 95% solvent A (water) and 5% solvent B (acetonitrile); then the ratio of solvent B was increased to 85% in 40 min followed by reducing solvent B to 5% in 50 min. This ratio was continued to 65 min for the next analysis with flow rate of 0.8 mL/min. All the standards were purchased from Sigma Chemical Company.

### 2.3. Determination of Total Phenolic Compounds Using Colorimetric Method

According to the determined total phenolic compounds using HPLC, the amounts of phenolic compounds in the* Poria* and* Gypsum* were very low or nondetectable. Since the phenolic compounds are the main antioxidant compounds which can reduce heat and oxidative stress in the animal tissues,* Poria* and* Gypsum* were not considered for further investigation using colorimetric method. The amounts of total phenolic compounds in the six other herb extracts were determined with the Folin-Ciocalteu reagent using the method of Spanos and Wrolstad [5]. Five ml of each extract (three replicates), 0.25 ml 1/10 dilution of Folin-Ciocalteu's reagent, and 0.2 ml of Na_2_CO_3_ (7.5%, w/v) were incubated at 45°C for 15 min. The absorbance of the mixtures was measured at 765 nm using a SPECTRAmax-PLUS384 UV-Vis spectrophotometer. Results were expressed as mg gallic acid equivalent per g of dry weight (mg GAE/g DW).

### 2.4. Determination of Antioxidant Activity

For determination of antioxidant activity of the samples, two methods, namely, 2,2′-azino-bis(3-ethylbenzothiazoline-6-sulfonic acid) (ABTS assay based on the method described by Tsai et al. [6]) and ferric reducing ability of plasma (FRAP assays according to the method developed by Benzie and Strain [7]), were used. Both methods have been completely described previously [8]. In the present study, for the ABTS assay, different concentrations of trolox (5 to 50 *μ*g/ml) were used to prepare the standard curve, and the results were expressed as Trolox Equivalents Antioxidant Capacity (TEAC) in the form of *μ*g Trolox/g sample; and for the FRAP assay, FRAP reagent was used as blank and the final absorbance of each sample was compared with those obtained from the standard curve made from 0 to 1000 *μ*mol/L ferric sulphate (FeSO4-7H2O).

### 2.5. LCMS/MS Analysis

A brief description for LCMS/MS method is as follows: Method: LCMS/MS = full scan with MS/MS data collection; ionization mode: Neg; column: Phenomenex Synergi Fusion 100 mm × 2.1 mm × 3 *μ*m; buffer A: water with 0.1% formic acid; buffer B: acetonitrile with 0.1% formic acid; rapid screening performed at 15 min run time using AB Sciex 5500QTrap LCMS/MS with Agilent 1290 series UHPLC; gradient run program: 5% B to 95% B from 0.01 min to 10.0 min, held for 2 min and back to 10% B in 0.1 min and reequilibrated for 3 min. MS settings and conditions were as follows: negative; voltage IS: 4500 V; source temperature: 500°C; desolvation gas: 40 psi; source gas: 40 psi; scan range: 100–1000* m/z* for full scan and 50–1000* m/z* for MS/MS scan.

### 2.6. Quantification of Total Carbohydrate in Form of Monomer Using HPLC

For determination of total monosaccharides, 100 mg of each dried sample was hydrolyzed using 10 mL of 4% sulfuric acid solution at 110°C for 6 h [according to Szambelan and Nowak [9] with minor modifications]. After hydrolysis, samples were centrifuged at 10000 ×g for 5 min, filtered through 0.22 *μ*m nylon syringe filters (Pall Gelman Laboratory, USA), and injected into the HPLC. Concentrations of MSC were assayed using HPLC (2690, Waters, USA) with a COSMOSIL Sugar-D column (250 × 4.6 mm i.d., 5 *μ*m). The mobile phase consisted of acetonitrile and water (80 : 20, v : v) with a flow rate of 1 mL/min and the column temperature of 35°C. A reflective index (RI) detector (2414, Waters, USA) was used for the detection of MSC in the detector sensitivity of 1024 and temperature of 30°C. The sample injection volume was 20 *μ*L, and the running time was 20 min. Arabinose, xylose, glucose, galactose, fructose, and mannose (Sigma-Aldrich, St. Louis, MO) were used as standard. In this method of sugar analysis by HPLC, glucose and galactose had the same retention time. This was the same for arabinose and xylose.

### 2.7. Determination of Fatty Acid Profile

Determination of fatty acid profile using Gas Chromatography (GC) followed the method described by Folch et al. [10] with minor modifications that were explained previously [11]. The fatty acid concentrations were expressed as percentage of the total identified peaks measured for each sample.

## 3. Results

### 3.1. Detection of Phenolic Compounds by HPLC

**Figure 1:**
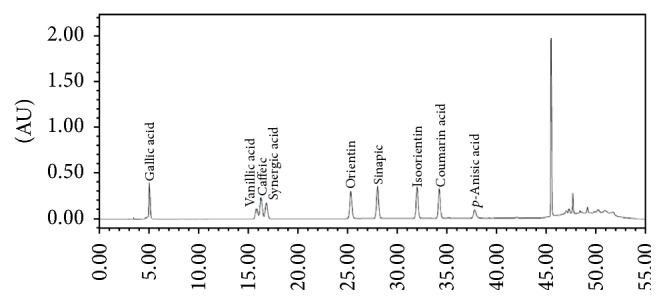
Chromatogram of phenolic compound mixture of the standards detected at 280 nm by RP-HPLC. Standards were gallic acid, vanillic acid, caffeic acid, synergic acid, orientin, isoorientin, sinapic acid, coumarin acid, and* p*-anisic acid.

The chromatogram of the nine phenolic standards (gallic acid, vanillic acid, caffeic acid, synergic acid, orientin, isoorientin, sinapic acid, coumarin acid, and* p*-anisic acid) is shown in Figure 1. Results of the concentration of the nine phenolic compounds in the herbal extract samples detected by HPLC (Table 1) showed that* Paeonia* contained the highest level of total phenolic compounds (17353 *μ*g/g DW) due to the high level of gallic acid (16336 *μ*g/g DW), while* Glycyrrhiza* had the second highest concentration of total phenolic compounds (12318 *μ*g/g DW) mainly due to the high level of p-anisic acid (9703 *μ*g/g DW), followed by* Mentha*,* Psoralea*,* Atractylodes*, and* Bupleurum* (6918, 5287, 3985, and 2595 *μ*g/g DW, resp.). Since the levels of total phenolic compounds in the* Poria* and* Gypsum* were very low or nondetectable, they were not included in our further investigations.

### 3.2. Determination of Total Phenolic Compounds by a Colorimetric Method and Antioxidant Activity by ABTS and FRAP Methods

Total phenolic compounds detected by a spectrophotometer [as gallic acid equivalents (GAE)] and antioxidant activity of the samples are presented in Table 2. Based on the colorimetric study,* Psoralea* had the highest level of phenolic compounds (17.4 GAE/g) and also the highest antioxidant activity measured using the ABTS and FRAP methods, followed by* Glycyrrhiza* (13.7 GAE/g) and* Paeonia* (11.2 GAE/g). The lowest phenolic content was detected in* Mentha* (3.6 GAE/g).

### 3.3. LCMS/MS Analysis of Herbal Extracts

Results of the LCMS/MS analysis of the six herbal extracts are shown in Table 3. Fourteen compounds were detected in* Atractylodes*, eight in* Paeonia*, seven in* Mentha*, six in* Glycyrrhiza*, and five in* Bupleurum*. Based on the library of LCMS/MS system that was used in the present study, no phenolic compound was detected in* Psoralea*.

### 3.4. Quantification of Total Sugars in Form of Monomer Using HPLC

The concentrations of total sugars in the samples after hydrolysis by 4% sulfuric acid are presented in Table 4.* Paeonia* contained the highest level of total sugars (62.38% of DM), mainly glucose and galactose.* Glycyrrhiza* contained 43.26 of monosaccharides (% DM) with glucose and galactose also having the greater values.* Bupleurum* contained the highest concentration of arabinose and xylose (10.28%), followed by glucose and galactose (10.13%). The lowest concentration of total sugars was detected in* Psoralea* (14.32%).

### 3.5. Fatty Acid Profile

Fatty acid profiles of the tested herbs are shown in Table 5.* Atractylodes* contained the highest levels of unsaturated fatty acids (84.54%) followed by* Glycyrrhiza* (78.87%),* Paeonia* (77.71%),* Bupleurum* (67.60%),* Mentha* (62.79%), and* Psoralea* (37.25%). In all samples, the percentage of unsaturated fatty acids was higher than that of saturated fatty acids apart from* Psoralea*. In the* Atractylodes*, 76.67% of fatty acids were in the form of polyunsaturated fatty acids with C18:2n-6 having a percentage of 70.94%.

## 4. Discussion

High environmental temperature in the tropics can cause huge economic losses in the poultry industry through reduced growth rate, egg production, and survival ability [12]. One of the most important biological adverse effects of heat stress is the oxidative injury (lipid peroxidation) and oxidative damage of proteins and DNA, which is caused by enhancement in formation of reactive oxygen species (ROS) [13]. It has been reported that there is a direct relationship between heat stress and the activation of antioxidant defense system in animals [14].

Alleviation of heat stress can be accomplished by increasing the antioxidant capacity of the animals after their dietary supplementations with antioxidant compounds. Moreover, promotion of gut health and intestinal barrier integrity can be achieved by using prebiotics alone or in combination with antioxidants. Supplementation with different compounds that possess antioxidant properties, including polyphenols, unsaturated fatty acids, and different prebiotic oligosaccharides have been already investigated with the intention to reduce the negative effects of heat stress in poultry [15, 16]. The use of natural antioxidant products derived from plants is preferred over chemical antioxidants due to food safety reasons [17]. Diao et al. [4] had recently reported that enrichment of the diet of laying hens with a mixture of eight Chinese herbal medicines (CHM) under heat stress condition significantly increased their egg production and immune responses. However, the active compounds of CHM responsible for alleviating the negative effects of heat stress were not investigated. This follow-up study aimed to provide some insights into the quantity and types of active compounds in the eight used CHM that might play an important role in heat stress reduction.

More than 10,000 individual phenolic compounds, known for their antioxidant activities, have been characterized from plant samples [18]. Results of our HPLC analysis showed that among the eight herbal samples tested, only six, namely,* Chai Hu* (*Bupleurum*),* Bai Zhu* (*Atractylodes*),* Bai Shao* (*Paeonia*),* Gan Cao* (*Glycyrrhiza*),* Bu Gu Zhi* (*Psoralea*), and* Bo He* (*Mentha*), contained considerable amounts of detectable phenolic compounds (gallic acid, vanillic acid, caffeic acid, synergic acid, orientin, sinapic acid, isoorientin, coumarin acid, and* p*-anisic acid). Results of the LCMS/MS detected many more different phenolic compounds from these six herbal samples. In addition, total phenolic compounds determination using the colorimetric method showed that, among the six herbs,* Psoralea* contained the highest concentrations of phenolic compounds, followed by* Glycyrrhiza* and* Paeonia*. Moreover,* Psoralea* also showed the highest antioxidant activity among the herbal extracts in the antioxidant assays.

Studies on feed and food supplementation of polyphenols showed increased antioxidant capacity in blood and in several organs [16]. Antioxidative effects of the dietary supplementation with phenolic compounds from various sources had been extensively studied in sheep [19], broilers [20–22], rabbits [23], and turkeys [24]. More recently, Akbarian et al. [25] examined the effects of dietary essential oils (rich in simple phenolic compounds) on mRNA levels of heat shock protein 70 (HSP70) and antioxidant enzymes, oxidative status, and meat oxidative stability of heat stressed broilers. They found that the enrichment with the dietary essential oils improved antioxidant defense against heat stress-induced changes. Improved antioxidant status of heat stressed broiler chickens using other phenolic compounds, such as flavonoids [26] and turmeric rhizome powder, rich in curcumin [27] is also reported. Prebiotics, such as galactooligosaccharides (GOS), xylooligosaccharides (XOS), mannan-oligosaccharides (MOS), and fructooligosaccharides (FOS), have also been reported to mitigate the adverse effects of heat stress. Varasteh et al. [15] reported that supplementation with dietary GOS resulted in a prevention of the negative heat stress-related changes in jejunum. Similarly, Hasan et al. [28] reported that prebiotic (MOS) enhanced growth in heat stressed broilers in comparison to the controls, but not in broilers under normal conditions.

The above-mentioned effects of prebiotics may be indirect or direct. The indirect effects include increase of the population of beneficial gut bacteria (probiotic bacteria) in the host animals which in turn improves their health and immune system response [29] contributing to overcoming of the adverse effect of heat stress. However, some prebiotics (e.g., inulin) which can be absorbed by liver and colon cells through pinocytosis [30, 31] act as antioxidants [17]. Epithelial gut cells can also assimilate other prebiotics such as soluble fructans and glucans [32]. In addition, prebiotics are also capable of exerting immune-modulating effects and can stimulate anti-inflammation responses [33], the first reaction against heat stress by the host's defense system. Hence, prebiotics could be a potential agent for reducing heat stress due to their immune-modulating effects and this hypothesis is receiving increasing interest [16].

A combination of several Chinese herbs rather than a single one is used to treat illness or stress. The reason is that a combination of several herbs could provide an expanded spectrum of active compounds. Our results showed that the order of herbal extracts according to the total amount of monosaccharides was* Paeonia*>* Glycyrrhiza*>* Bupleurum*>* Atractylodes*>* Mentha*>* Psoralea*, while that for the level of unsaturated fatty acid was* Atractylodes*>* Glycyrrhiza*>* Paeonia*>* Bupleurum*>* Mentha*>* Psoralea*. And for all the herbs, apart from the* Psoralea*, the amount of unsaturated fatty acids was higher than saturated fatty acids. Since unsaturated fatty acids, especially omega 3 series, are known for their antioxidant activity [34], the high levels of unsaturated fatty acids indicate possible high antioxidative efficacy of the herbs examined in this study.

## 5. Conclusion

For the first time, this study provides qualitative and quantitative information on the phenolic compounds, prebiotics, and fatty acids (known for their antioxidant capability) of several traditional herbs which had been shown to effectively reduce heat stress in layer hens. Although the use of CHM for treatment of various illnesses in human subjects has been used for many thousands of years, their use as “green” medications for livestock industry is rather recent, especially with increasing interest as they could possibly serve as alternatives for antibiotics to reduce stress and promote the growth in commercial livestock and poultry production. It must be emphasized that the current data is not exhaustive but could serve as a reference for future studies on similar subject. Based on the results of the present study,* Psoralea*,* Glycyrrhiza*, and* Paeonia* had high phenolic compounds while* Glycyrrhiza*,* Bupleurum*, and* Paeonia* contained higher levels of prebiotics. Content of unsaturated fatty acids was higher in* Atractylodes*,* Glycyrrhiza*, and* Paeonia*. However, the actual efficacy of these herbs, singly or as mixture, to alleviate heat stress requires further* in vivo* investigations. The importance of* Poria* and* Gypsum* that contain very low or nondetectable levels of phenolic compounds in the mixture of CHM in reducing heat stress needs further investigation.

## Figures and Tables

**Table 1:** Phenolic compounds of eight herbal medicine extracts determined using HPLC (*µ*g/g DW).

Phenolic compound	*Bai Zhu* (*Atractylodes*)	*Bai Shao* (*Paeonia*)	*Chai Hu* (*Bupleurum*)	*Bo He* (*Mentha*)	*Bu Gu Zhi* (*Psoralea*)	Fu Ling (*Poria*)	*Gan Cao* (*Glycyrrhiza*)	*Shi Gao* (*Gypsum*)
Gallic acid	2.52	16336	58	50	180	3	64	ND
Vanillic acid	561	226	885	836	1111	ND	362	ND
Caffeic acid	1413	56	229	2905	183	17	76	ND
Synergic acid	276	94	62	620	339	ND	45	ND
Orientin	67	119	53	1028	579	ND	224	ND
Sinapic acid	369	92	475	40	4	ND	1718	ND
Isoorientin	184	226	51	22	2579	ND	101	ND
Coumarin acid	896	29	679	210	35	ND	25	ND
*p*-Anisic acid	217	175	103	1207	277	24	9703	ND
Total	3985	17353	2595	6918	5287	44	12318	—

Values are mean of three replicates; ND: not detected.

**Table 2:** Total phenolic content and antioxidant activity of CHM samples.

Herbal medicine	Total phenolic content (mg gallic acid equivalents, GAE)/g	TEAC (*μ*mol of trolox equivalents, TE)/g	FRAP (*µ*mol Fe^2+^/g)
*Bai Zhu *(*Atractylodes*)	5.78	53.4	28.96
*Bai Shao *(*Paeonia*)	11.22	89.67	142.23
*Chai Hu *(*Bupleurum*)	4.71	41.82	32.58
*Bo He *(*Mentha*)	3.6	27.5	28.91
*Bu Gu Zhi* (*Psoralea*)	17.4	91.67	232.98
*Gan Cao *(*Glycyrrhiza*)	13.7	77.48	167.65

Values are mean of three replicates.

**Table 3:** Phenolic and flavonoid compounds in the herbal medicine samples detected by LCMS/MS.

Name	Name of compound
*Bai Zhu *(*Atractylodes*)	3,30-Di-O-methyl ellagic acid derivatives
	3,30-Di-O-methyl ellagic acid
	Caffeoylquinic acid derivatives
	Cinnamic acid derivatives
	Ferulic acid quinic acid conjugate
	Gluconic acid
	Methoxyflavone derivatives
	Petunidin-3-O-(4 coumaroyl)-rutinose-5-O-glucose)
	Phenylvaleric acid
	Quinic acid
	Rhamnoside flavanoids 2
	Rhamnoside flavanoids
	Rutinoside flavanoids
	Tannic acid

*Bai Shao *(*Paeonia*)	Apigenin derivatives
	Quinic acid
	Gallic acid derivatives
	Methoxyflavone derivatives
	Myricetin-3-O-galactose dimer
	Rhamnoside flavanoids
	Rutinoside flavonoids
	Tannic acid

*Bo He *(*Mentha*)	Caffeoylquinic acid isomer
	Protocatechuic acid derivatives
	Rosmarinic acid derivative
	Sagerinic acid
	Salvianolic acid A
	Salvianolic acid B isomer
	Tannic acid

*Gan Cao *(*Glycyrrhiza*)	Apigenin 7-O-(6′′-dihydrogalloyl) isomer
	Apigenin-6,8-di-C-*β*-D-glucopyranoside isomer
	Caffeoyl glucose
	Gluconic acid
	Phenylvaleric acids
	P-Hydroxycinnamoyl derivative

*Chai Hu *(*Bupleurum*)	3,30-di-O-methyl ellagic acid
	15,16-dihydroxy-9Z,12Z-octadecadienoic acid
	16-alfa-o-Methylneoquassin metabolite
	Methoxyflavone derivatives
	Tannic acid

*Bu Gu Zhi* (*Psoralea*)	Not detected

**Table 4:** Total sugar (% of dry matter) in form of monosaccharide in the samples determined using HPLC.

Herbal medicine	Arabinose and xylose	Fructose	Mannose	Glucose and galactose	Total
*Bai Zhu *(*Atractylodes*)	10.28	3.26	3.64	7.08	24.26
*Bai Shao *(*Paeonia*)	6.5	ND	0.58	55.3	62.38
*Chai Hu *(*Bupleurum*)	11.79	9.13	2.85	10.13	33.9
*Bo He *(*Mentha*)	10.15	ND	2.7	8.93	21.78
*Bu Gu Zhi* (*Psoralea*)	ND	5.1	3.7	5.52	14.32
*Gan Cao *(*Glycyrrhiza*)	7.67	4.73	ND	30.86	43.26

Values are mean of three replicates; ND: not detectable.

**Table 5:** Fatty acid profiles of herbal medicine extracts (based on %).

Herbal medicine	C14:0	C16:0	C16:1	C18:0	C18:1n-9	C18:2n-6	C18:3n-6	C18:3n-3	TSFA	TUFA	TMUFA	Total PUFA	TUFA:TSFA
*Bai Zhu* (*Atractylodes*)	0.00	13.89	0.00	1.57	7.87	70.94	0.00	5.73	15.46	84.54	7.87	76.67	5.47
*Bai Shao* (*Paeonia*)	0.00	19.67	0.36	2.26	22.58	49.74	0.00	5.39	22.29	77.71	22.58	55.13	3.49
*Chai Hu* (*Bupleurum*)	0.00	26.56	0.83	5.01	23.17	37.99	1.86	4.59	32.40	67.60	23.17	44.44	2.09
*Bo He* (*Mentha*)	1.71	28.98	1.12	5.40	21.75	14.56	1.82	24.67	37.21	62.79	21.75	41.05	1.69
*Bu Gu Zhi* (*Psoralea*)	0.00	9.99	0.00	52.76	9.50	19.44	0.58	7.73	62.75	37.25	9.50	27.75	0.59
*Gan Cao* (*Glycyrrhiza*)	0.00	17.63	0.15	3.35	18.01	51.62	0.00	9.25	21.13	78.87	18.01	60.87	3.73

Values are mean of three replicates; C14:0, myristic acid; C16:0, palmitic acid; C16:1, palmitoleic acid; C18:0, stearic acid; C18:1n-9, oleic acid; C18:2n-6, linoleic acid; C18:3n-6, *γ*-linolenic acid; C18:3n-3, *α*-linolenic acid; TSFA, total saturated fatty acids; TUFA, total unsaturated fatty acids; TMUFA, total monounsaturated fatty acids; TPUFA, total polyunsaturated fatty acids; TUFA:TSFA, unsaturated fatty acids: saturated fatty acids ratio.
